# Research Advances in Adenomyosis-Related Signaling Pathways and Promising Targets

**DOI:** 10.3390/biom14111402

**Published:** 2024-11-04

**Authors:** Hongyu Zhang, Chaoming Li, Wenyan Li, Wenhu Xin, Tiansheng Qin

**Affiliations:** 1 Department of Gynecology, The Second Hospital & Clinical Medical School, Lanzhou University, Lanzhou 730000, China; 2 Departmemt of Urology, The First People’s Hospital of Longnan, Longnan 742500, China

**Keywords:** adenomyosis, pathophysiology, molecular medicine, pathways, targets

## Abstract

Adenomyosis is a benign gynecological condition characterized by the proliferation of the endometrial stroma and glands into the myometrium, uterine volume enlargement, and peripheral smooth muscle hypertrophy. The typical clinical symptoms include chronic pelvic pain, abnormal uterine bleeding, and subfertility, all of which significantly impact quality of life. There are no effective prevention or treatment strategies for adenomyosis, partly due to a limited understanding of the pathological mechanisms underlying the initiation and progression of the disease. Given that signaling pathways play a crucial role in the development of adenomyosis, a better understanding of these signaling pathways is essential for identifying therapeutic targets and advancing drug development. The occurrence and progression of adenomyosis are closely linked to various underlying pathophysiological mechanisms, including proliferation, migration, invasion, fibrosis, angiogenesis, inflammation, oxidative stress, immune response, and epigenetic changes. This review summarizes the signaling pathways and targets associated with the pathogenesis of adenomyosis, including CXCL/CXCR, NLRP3, NF-κB, TGF-β/smad, VEGF, Hippo/YAP, PI3K/Akt/mTOR, JAK/STAT, and other relevant pathways. In addition, it identifies promising future targets for the development of adenomyosis treatment, such as m6A, GSK3β, sphks, etc.

## 1. Introduction

Adenomyosis was probably first described by Babes in 1882. The current definition was provided by Bird in 1972 [1], describing it as a benign condition in which endometrial tissue invades the myometrium, resulting in diffuse enlargement of the uterus [2]. This condition is characterized by the infiltration of the myometrium by endometrial glands and stroma, leading to chronic pelvic pain, abnormal uterine bleeding, and infertility, which significantly affect women’s reproductive health. But nearly one-third of asymptomatic patients remain undiagnosed [3]. One reason for this is the lack of consistent diagnostic criteria and a comprehensive understanding of the progression of the pathological mechanisms. In recent years, non-invasive transvaginal ultrasound (TVUS) and magnetic resonance imaging (MRI) have demonstrated advantages in the diagnosis of adenomyosis [4,5]. Nevertheless, the absence of effective treatment and prevention strategies for adenomyosis remains a significant challenge in women’s health. Previous clinical treatment approaches have focused on hysterectomy, uterus sparing surgery, and medication [6]. However, these strategies carry potential adverse side effects, including infertility and the risk of recurrence following the discontinuation of medication, which pose significant challenges to effective treatment. Therefore, there is an urgent need to explore new treatment strategies for adenomyosis to improve patient outcomes and quality of life.

The pathogenesis of adenomyosis remains unknown, and the most widely accepted mechanisms are thought to be invagination and metaplasia. Tissue injury and repair (TIAR) mechanisms are believed to be at the origin of the condition [7], where various factors, such as uterine hyperperistalsis, pregnancy, iatrogenic injury, and other factors, lead to micro-injuries at the junctional zone (JZ) between the endometrium and the muscle layer of the uterus. This results in an increase in the local release of estrogen and inflammatory mediators, which regulate cell migration, invasion, and proliferation, promoting fibrosis and neovascularization, thereby accelerating the occurrence of adenomyosis. Epigenetic defects, alterations in genes, the overexpression of aromatase, progesterone resistance, and excessive inflammasome activity are all thought to increase the risk of this disease [8]. Long-term exposure to non-steroidal anti-inflammatory drugs (NSAIDs) and 17β-ethinylestradiol mixtures at environmental doses can intergenerationally affect uterine physiology, inducing adenomyosis in mice [9].

Adenomyosis is a benign disease, but it exhibits characteristics typically associated with malignancy, including enhanced invasive ability, metastasis, angiogenesis, and evasion of apoptosis. Epidemiological data indicate that adenomyosis increases the risk of various cancers in women, including ovarian, endometrial, breast, and colorectal cancers [10]. Consequently, recent research on adenomyosis has concentrated on the disease’s pathophysiology and molecular biology. There is hope that new strategies for the clinical treatment of adenomyosis can be developed by targeting critical signaling pathways and key molecules.

## 2. Hormone-Related Signaling Pathways

### 2.1. Estrogen

Adenomyosis is a steroid hormone-dependent disease characterized by increased local estrogen (E2) levels and progesterone (P4) resistance. Key features include significantly elevated expression of estrogen receptors, enhanced aromatase activity, and decreased expression of 17β-hydroxysteroid dehydrogenase type 2, leading to an imbalance and excessive accumulation of local estrogen metabolites [11]. Higher aromatase activity in adenomyosis may result in increased local estrogen levels, exacerbating symptoms [12,13]. Increased estrogen induces the expression of oxytocin and its receptors, enhances uterine hyperperistalsis [12], activates TIAR, and ultimately contributes to the development of adenomyosis. Additionally, the rise in estrogen levels also induces epithelial–mesenchymal transition (EMT), facilitating the invasion of the endometrium into the uterine muscle layer, which stimulates the occurrence of adenomyosis. Activation of the Wnt/β-catenin signaling pathway often induces EMT and cell proliferation [14], playing a role in the pathogenesis.

Increased local estrogen stimulates the activity of downstream cyclooxygenase-2 (COX-2), accelerates the synthesis of prostaglandin E2 (PGE2), and contributes to the onset of the disease. COX-2 serves as not only a therapeutic target for dysmenorrhea but is also closely associated with inflammation and angiogenesis [15]. Elevated estrogen levels enhance the expression of COX-2, and a direct correlation exists between COX-2 levels and interleukin (IL)-6/8 [16], thereby mediating the inflammation response. Further research indicates that the selective COX-2 inhibitor celecoxib reduces estrogen production [17], as well as fibrosis and EMT in the uterus, effectively alleviating the disease. Notably, Wnt/β-catenin signaling is altered in mouse models of adenomyosis [14], and COX-2 expression is positively correlated with β-catenin levels in the endometrium of adenomyosis, suggesting that COX-2 may be involved in the pathogenesis of adenomyosis through the Wnt/β-catenin signaling pathway [15]. Therefore, targeting COX-2 and inhibiting its overexpression can alleviate dysmenorrhea symptoms while also reducing local estrogen accumulation, inflammation, fibrosis regulation, and EMT inhibition.

The overexpression of the Ras homolog family member A/Rho-associated coiled-coil-containing protein kinase (RhoA/ROCK) in adenomyosis cells has been linked to elevated local estrogen expression [18,19]. In a murine model of endometriosis, excessive estrogen has been shown to enhance phosphorylated ERK and stimulate the expression of RhoA and ROCK 1/2 [20], thereby facilitating EMT and proliferation. In summary, heightened estrogen levels can activate ERK signaling, upregulate the RhoA/ROCK pathway, and promote EMT and proliferation. Conversely, reducing local estrogen levels and suppressing the RhoA/ROCK pathway may alleviate focal establishment and reverse EMT. The RhoA/ROCK pathway presents a promising molecular target for local high doses of antiestrogens.

### 2.2. Progestogens

Progestogen signaling is essential for counteracting estrogen-induced endometrial proliferation and promoting decidualization [21]. P4 resistance is considered a crucial element in endometrial diseases. Studies have found that progesterone can stimulate the endometrial secretion of prolactin (PRL), and the signaling pathway mediated by the significant upregulation of PRL and its receptor PRLR may enhance uterine peristalsis in adenomyosis [11], facilitating the establishment of lesions. In steroid hormone-dependent diseases, P4 resistance is associated with the loss or reduction in downstream target mitogen-inducible gene 6 (MIG-6). A loss of MIG-6 leads to the activation of the tyrosine kinase receptor 2 (ErbB2)-ERK signaling pathway, promoting the proliferation of uterine epithelial cells [22,23]. Additionally, targeting ErbB2 can reverse the development of endometrial hyperplasia, infertility, and endometriosis lesions caused by reduced MIG-6. Nevertheless, research on MIG-6 and ErbB2 in adenomyosis is lacking and more research is needed.

Second-generation gene sequencing (NGS) indicates that adenomyosis is a condition associated with mutations in the KRAS gene [8]. KRAS mutations not only induce high methylation of the progesterone receptor, affecting P4 resistance, but also enhance tissue invasion and growth, thereby promoting lesion formation. Future research is needed to explore the upstream and downstream molecular pathways associated with KRAS gene mutations in adenomyosis, such as phosphatidylinositol 3-kinase/phosphoinositide dependent kinase 1/Protein Kinase B (PI3K/PDK1/AKT) and Rapidly Accelerated Fibrosarcoma/Mitogen-Activated Protein Kinase/extracellular signal-regulated kinase (RAF/MEK/ERK) pathways [24,25], and find precision therapy targeting KRAS mutations. In addition to KRAS, mutations in the AT-rich interaction domain 1A (ARID1A) gene activate the PI3K signaling pathway [26,27], driving cell migration and invasion and promoting the occurrence of EMT.

### 2.3. N6-Methyladenosine

Epigenetic modifications, including DNA methylation, histone methylation, acetylation, and RNA methylation, play an important role in regulating human physiology and invasive diseases. Most studies have focused on the role of DNA methylation. The overexpression of estrogen receptor beta (ERβ) leads to reduced expression of the progesterone receptor, while high methylation of the progesterone receptor beta (PRβ) promoter is a cause of progesterone resistance. RNA methylation has also been implicated in reproductive system diseases, with decreased levels of methyltransferase-like 3 (METTL3) and N6-methyladenosine (m6A) observed in adenomyosis [28,29]. This regulation affects downstream proliferative factors, such as insulin-like growth factor-1 (IGF1) and D-dopachrome tautomerase (DDT), leading to the activation of the downstream AKT/mammalian target of rapamycin (mTOR) signaling pathway, which plays a crucial role in epithelial proliferation and cell migration. METTL3-dependent m6A methylation also regulates the estrogen–progesterone balance [30] and activates the Wnt signaling pathway, inducing EMT and angiogenesis. A study also found that a loss of METTL3 results in decreased expression of the progesterone receptor (PGR) protein, and that the METTL3-PGR axis plays a role in ovarian steroid-dependent diseases [31]. In conclusion, we speculate that m6A regulates the expression of METTL3 and downstream genes, activating AKT to affect cell proliferation and migration and thereby promoting the establishment of adenomyosis [32].

It is worth noting that epigenetic modifications are reversible, and an increasing number of drugs targeting epigenetic changes are under development. Epigallocatechin gallate (EGCG), a catechin found in green tea, has been shown to influence proliferation, angiogenesis, inflammation, and fibrosis, while also inhibiting or attenuating EMT in treatment strategies for reproductive system diseases [33]. In a study of mouse adenomyosis, EGCG emerged as a promising treatment strategy that suppressed myometrial infiltration, improved generalized hyperalgesia, and reduced uterine contractility, while elevating the expression of the progesterone receptor [34,35].

## 3. Inflammation-Related Signaling Pathways

### 3.1. CXCL/CXCR

Abnormal expression of continuous cell adhesion and chemokine ligand/receptor (CXCL/CXCR) signaling pathways in the endometrium drives the migration of endometrial cells into the uterine muscle layer [36,37], leading to the development of adenomyosis (Table 1). Among the chemokines, CXCL8 (IL-8) plays a crucial role in inflammation, immunity, fibrosis, angiogenesis, hematopoiesis, and cell proliferation and differentiation [38,39]. Compared to healthy individuals, the levels of IL-8 are significantly elevated in patients with adenomyosis [40]. Additionally, the expression of IL-8 receptors, CXCR1 and CXCR2, is increased in adenomyosis lesions, suggesting that IL-8 and its receptors may be involved in the pathogenesis of adenomyosis [41]. Data comparing the levels of IL-8 before and after treatment for adenomyosis reveal a significant decrease post-treatment, providing further evidence of the role of IL-8 in disease onset [42]. It is noteworthy that previous studies have indicated that IL-8 plays a similar role in the pathogenesis of both adenomyosis and endometriosis. Recent findings in a baboon model of chronic inflammatory disease have shown that upregulation of IL-8 was observed. Treatment with IL-8 antibodies or inhibitors effectively reduced lesion size and alleviated fibrosis and adhesions [43]. We propose that IL-8 may be a promising therapeutic target. Other studies have reported that CXCL12 and its receptor CXCR4 are significantly elevated in the endometrium of patients with adenomyosis [44]. The CXCL12/CXCR4 signaling pathway is involved in pathological mechanisms such as inflammation, fibrosis, EMT, and angiogenesis, and it has also been identified as a potential target for anti-fibrosis therapy [45]. Therefore, inflammatory cytokine signaling pathways not only maintain the balance between pro-inflammatory and anti-inflammatory factors but also participate in various pathological and physiological mechanisms, such as tissue fibrosis and EMT. Future research is needed to explore whether other inflammatory cytokine pathways play a role in the regulation of adenomyosis. For example, IL-6, IL-10, and IL-22 show altered expression in adenomyosis lesions. However, the specific signaling pathways and the mechanisms underlying these changes still require further elucidation [46].

### 3.2. NF-κB

NF-κB is an important transcriptional regulator in eukaryotic cells, mainly regulating inflammatory responses. The Toll-like receptor 4 (TLR4)/MyD88/nuclear factor-kappa B (NF-κB) signaling pathway has been found to promote the secretion of various cytokines and growth factors in adenomyosis, leading to inflammatory responses and immune system alterations [40,58], thereby contributing to disease onset. In studies of adenomyosis, low expression of histone deacetylase 3 (HDAC3) has been negatively correlated with the degree of fibrosis, and reduced HDAC3 expression disrupts the NF-κB pathway, impairing the resolution of inflammation and endometrium repair and ultimately exacerbating the disease [59]. HDAC3 has been proposed as a novel pharmacological target, with inhibitor therapy shown to be effective in treating various diseases. For example, in chronic kidney disease, there is a positive correlation between HDAC3 expression and fibrosis, and HDAC3 inhibition has been effective in reducing disease progression [60,61]. However, this contrasts with findings in adenomyosis and endometriosis [59,62], probably due to the differential regulation of HDAC3 expression in different organs, or the limited number of HDAC3 studies in adenomyosis, which are currently only available from mouse models. Further research is clearly warranted to clarify these mechanisms.

Beyond its role in inflammatory responses, the NF-κB signaling pathway also regulates tissue fibrosis, EMT, and cell proliferation, migration, and invasion. Future research should focus on the intricate cross-talk between different molecular networks within this pathway. For instance, NF-κB/β-catenin [63], NF-κB/p65 [64,65], NF-κB/Stat3 [64], and NF-κB/apoptosis signaling [66] are interconnected in the pathogenesis and treatment of adenomyosis.

### 3.3. NLRP3

The nucleotide-binding domain, leucine-rich-repeat family, pyrin-domain-containing 3 (NLRP3) forms an inflammasome complex with caspase-1, which induces the production of the pro-inflammatory cytokine IL-1β, thereby enhancing immune responses and promoting pyroptosis [67]. In the inflammatory disease adenomyosis, downregulation of the apoptosis-related gene 19 (GRIM19) activates the NLRP3 signaling pathway, leading to macrophage pyroptosis and increased secretion of IL-1β [68]. This cascade contributes to disease progression. Furthermore, increased expression of ERβ can activate the inflammasome, thereby promoting cell proliferation and invasion [11,69]. NLRP3 is also upregulated in endometriosis, and inhibiting NLRP3 levels can reduce IL-1β levels in stromal cells [70]. Consequently, in estrogen-driven inflammatory diseases, NLRP3 activation may facilitate disease development. A recent article has explored the potential of NLRP3 as a therapeutic target for adenomyosis [71]. The study reported elevated expression of NLRP3 inflammasome components in both ectopic and eutopic endometrial tissues of patients with adenomyosis. Furthermore, the use of an NLRP3 inhibitor in a mouse model of adenomyosis resulted in reduced migration and invasion of endometrial cells, contributing to the alleviation of the disease. Thus, targeting NLRP3 to inhibit the activation of the NLRP3 inflammasome may represent a promising therapeutic strategy for the management of adenomyosis.

## 4. Fibrosis-Related Signaling Pathways

### 4.1. TGF-β/Smad

Growing evidence suggests that fibrosis plays a crucial role in the pathogenesis of adenomyosis. Tissue damage repair, ECM deposition, EMT, fibroblast-to-myofibroblast transdifferentiation (FMT), smooth muscle metaplasia (SMM), and immune responses are all key contributors to the fibrotic process in adenomyosis [72,73].

**Figure 1:**
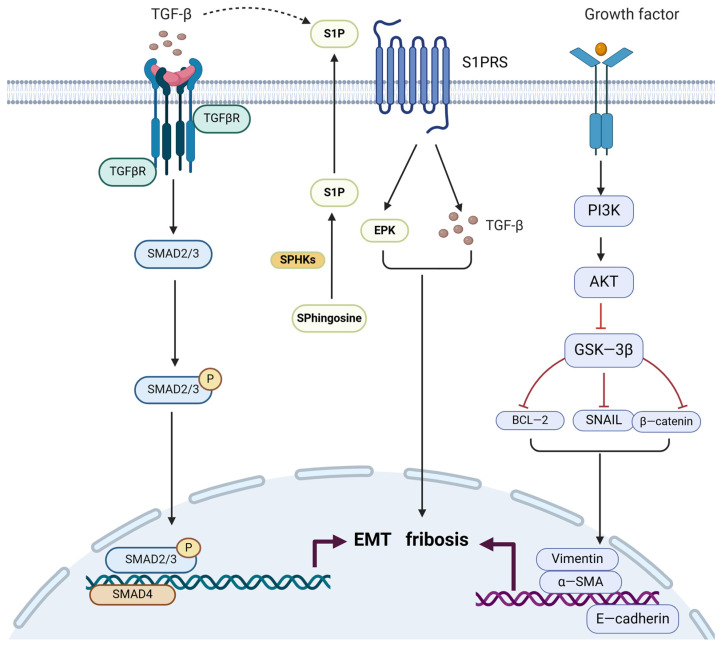
The fibrosis-related signaling pathways mentioned above. Created in BioRender. Hy, Z. (2024) https://BioRender.com/b09f513 (accessed on 1 November 2024). Legend: the red lines represent inhibitory effects. PI3K, phosphoinositide-3 kinase; GSK-3β, Glycogen synthase kinase-3; SphKs, sphingosine kinases; S1P, Sphingosine 1-phosphate; α-SMA, α-smooth muscle actin.

Transforming growth factor-beta (TGF-β) is a major driver of fibrosis [74], and Smad is a downstream effector in the TGF-β signaling pathway. Increased secretion of TGF-β activates Smad through the TGF-β receptor (TGFBR) 1/2 (Figure 1), initiating downstream gene expression and promoting fibrotic lesions [75]. Previous studies have shown that TGF-β1 levels are significantly increased in patients with adenomyosis, and anti-TGF-β1 treatment was found to reduce uterine collagen expression, thereby inhibiting fibrosis associated with adenomyosis. Beyond the TGF-β/Smad signaling pathway, TGF-β1 plays a critical role in fibrosis, leading to suggestions that it could serve as a therapeutic target for adenomyosis. However, some studies have reported that TGF-β1 levels are not elevated in patients with adenomyosis, contradicting previous findings [76]. These discrepancies may be due to the fact that much of the research on anti-TGFβ has focused on mouse models of adenomyosis, leading to species differences and limited human data, which introduces variability. Recent findings indicate that TGF-β2 is significantly elevated in both mouse models and human validation data regarding adenomyosis, and it induces disease onset through β-catenin activation. This suggests that TGF-β2 may be a promising therapeutic target for adenomyosis [14]. While the expression levels of TGF-β1 and TGF-β2 in adenomyosis remain uncertain, the critical role of the TGF-β signaling pathway in the pathogenesis of adenomyosis and the potential efficacy of TGF-β inhibitors are widely recognized.

In the treatment of liver fibrosis, the overexpression of the tyrosine kinase B antibody (TrkB) has been shown to inhibit the activation of the TGF-β/Smad signaling pathway [75], potentially exerting anti-fibrotic effects. TrkB protein expression is significantly increased in the secretory endometrium of patients with adenomyosis, where it activates the PI3K/AKT pathway, and is positively correlated with serum CA125 levels and dysmenorrhea [77]. However, whether TrkB in adenomyosis is involved in the regulation of the TGF-β/Smad signaling pathway and the alleviation of fibrosis remains unknown and requires further investigation.

### 4.2. AKT/GSK-3β

Glycogen synthase kinase-3 (GSK-3) plays a critical role in organ tissue fibrosis. The GSK-3 family consists of two subtypes, GSK-3α and GSK-3β. When tissue damage occurs (Figure 1), the inhibition of GSK-3β affects the expression of downstream target genes, including transcription factors such as snail, Bcl-2, and β-catenin. This leads to a reduction in levels of α-smooth muscle actin (α-SMA) and vimentin, as well as decreased collagen synthesis and accumulation, thereby inhibiting fibrosis [78].

Key downstream targets of GSK-3β, including vimentin [73], Bcl-2 [66], snail [63], and α-SMA [73], are highly expressed in adenomyosis lesions. Research indicates that aberrant activation of β-catenin and the overexpression of Bcl-2 contribute to the pathogenesis of adenomyosis [63,66], suggesting their potential as therapeutic targets for this condition. Reducing the expression of these proteins in adenomyosis lesions could offer an effective treatment strategy. Furthermore, as a common upstream signaling molecule, GSK-3β may influence the treatment of adenomyosis through the modulation of its signaling pathway. The PI3K/AKT signaling pathway is widely present in cells and plays a crucial role in regulating GSK-3β. As the most prevalent proliferation-associated pathway, PI3K/AKT is activated in adenomyosis. Based on network pharmacology, it has been suggested that inhibiting the PI3K/AKT signaling pathway to reduce Bcl-2 expression is a promising therapeutic strategy [79]. In summary, GSK-3β serves as a key link between proteins such as PI3K/AKT and Bcl-2, making it a potential target for the treatment of adenomyosis.

### 4.3. SphKs/S1P/S1PRs

Sphingosine 1-phosphate (S1P) is often involved in fibrosis and inflammatory diseases, primarily by activating the local secretion of TGF-β and growth factors through the S1P signaling pathway. This activation stimulates the synthesis of IL-1β and TGF-β, leading to EMT and tissue fibrosis [80]. Additionally, S1P is also a pro-angiogenic factor, and its inhibitors can suppress angiogenesis [81]. Previous studies have demonstrated that an imbalance in S1P signaling mediates the overexpression of TGF-β in endometriosis, contributing to fibrosis and EMT. The sphingosine kinase (SphK)/S1P signaling pathway is considered a potential therapeutic target as it promotes EMT and is associated with the ERK signaling pathway in conditions like endometriosis [82,83,84]. In 2022, the first evidence of an S1P signaling imbalance related to fibrosis was discovered in adenomyosis [85]. Therefore, the SphK/S1P/S1P receptor (S1PR) signaling pathway mediates the expression and activation of TGF-β and EPK signaling (Figure 1), inducing the progression of fibrosis and EMT. Inhibiting this pathway could potentially slow the growth and fibrosis of ectopic lesions. In conclusion, S1P and its mediated signaling pathways represent promising new therapeutic targets for adenomyosis.

## 5. Angiogenesis Signaling Pathways

### 5.1. Chemokines

**Figure 2:**
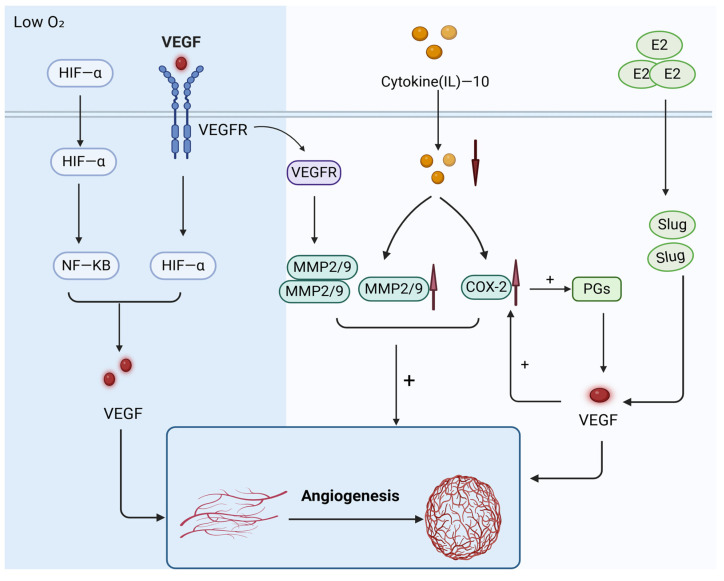
Angiogenesis and cell proliferation signaling pathways. Created in BioRender. Hy, Z. (2024) https://BioRender.com/s09j901 (accessed on 31 October 2024). Legend: the red arrows pointing upwards indicate an increase, while the red arrows pointing downwards signify a decrease in the variable. PGs, prostaglandins; HIF-α, hypoxia-inducible factor-1α.

The increased expression of angiogenic markers and microvessel density (MVD), along with the decreased expression of anti-angiogenic markers in individuals with adenomyosis compared to those without the condition, suggest that angiogenesis plays a significant role in the pathogenesis of adenomyosis (Figure 2). A 2019 systematic review found that common vascular and pro-angiogenic markers are upregulated in the ectopic endometrium, while anti-vascular factor expression is downregulated [46].

Chemokines are key regulators of both inflammation and angiogenesis. IL-10, an anti-angiogenic factor, inhibits angiogenesis mainly by downregulating COX-2 and matrix metalloproteinase (MMP)-2/9 [47]. However, IL-10 is significantly downregulated in adenomyosis [48], resulting in increased angiogenesis. A 2016 study proposed that vascular endothelial growth factor (VEGF) and CXCL1 are involved in the epithelial expression of the endometrium in adenomyosis. VEGF can induce CXCL1 expression in human endometrial epithelial cells through the VEGFR, p47 phox NADPH oxidase, and NF-κB signaling pathways, promoting the migration of vascular endothelial cells [86].

### 5.2. E2/VEGF

Estrogen (E2) is recognized as a pro-angiogenic factor that induces VEGF expression in endometrial epithelial cells, thereby promoting the development of adenomyosis. In a mouse model, researchers proposed that the E2-Slug-VEGF signaling pathway plays an important role in angiogenesis related to adenomyosis. Elevated E2 levels enhance angiogenesis by upregulating VEGF expression in both endometrial epithelial and endothelial cells. Studies have shown that targeting E2 and VEGF can effectively reduce angiogenesis in adenomyosis [87]. Additionally, there is a positive correlation between COX-2 and VEGF. The excess prostaglandins synthesized by COX-2 upregulate VEGF expression, which subsequently stimulates COX-2 expression in the endometrium [88].

### 5.3. HIF-α/VEGF

Previous studies have shown that VEGF expression is significantly increased in adenomyosis lesions, and that VEGF levels are proportional to hypoxia-inducible factor-1α (HIF-α), which is important in angiogenesis [89]. Angiogenesis is typically induced by hypoxia, which activates the NF-κB pathway through HIF-α expression, leading to the upregulation of the VEGF gene and influencing neovascularization [90]. Additionally, angiogenesis may be associated with oxidative stress, as evidenced by markers of angiogenesis induced by oxidative stress, such as HIF and VEGF expression. Beyond promoting angiogenesis, VEGF activates VEGF receptor 2 (VEGFR-2) to enhance cell proliferation and migration [91]. VEGF expression correlates positively with MMP-2 and MMP-9 [92], and inhibiting MMP-9 levels can suppress cancer cell proliferation, migration, and invasion and EMT [93]. NF-κB plays an important role in the pathogenesis of adenomyosis by regulating VEGF expression, suggesting that their interaction may be critical for disease progression and symptom development.

These findings suggest that inhibiting the activation of the NF-κB signaling pathway, reducing the expression levels of angiogenic factors, and suppressing MMP-9 may be promising in the treatment of adenomyosis. However, melatonin therapy has shown potential by inhibiting the NF-κB pathway while simultaneously increasing VEGF expression [66], effectively alleviating the disease. Therefore, effective treatment of adenomyosis may benefit from a comprehensive, integrative approach rather than focusing on a single therapeutic target or pathway.

## 6. Cell Proliferation, Migration, and Invasion Signaling Pathways

### 6.1. PI3K/AKT/mTOR

Adenomyosis is a benign gynecological disease, but it exhibits characteristics of malignant tumor proliferation and invasion, suggesting a potential association between adenomyosis and tumor-associated signaling pathways. In tumors, the PI3K/AKT/mTOR signaling pathway is most commonly activated [94]. The tumor suppressor phosphatase and tensin homolog (PTEN) gene physiologically inhibits the activation of the PI3K/AKT/mTOR pathway [95]. The PI3K/AKT signaling pathway is also activated in adenomyosis [79]. Within ectopic endometrial tissue in adenomyosis, LINC-ROR has been proposed to activate the PI3K/Akt signaling pathway, with research indicating that the expression of LINC-ROR and AKT is inversely related to PTEN levels [96]. Moreover, PTEN expression is downregulated in patients with adenomyosis [97]. This reduction in PTEN suggests that the activation of the PI3K/Akt/mTOR signaling pathway may lead to enhanced proliferation and invasion of endometrial cells. Treatment with progestins, such as Dienogest, downregulates the mTOR signaling pathway and promotes cell apoptosis [98].

Therefore, one therapeutic strategy for intervening in the development and progression of adenomyosis involves inhibiting the excessive activation of the PI3K/AKT/mTOR signaling pathway, thereby regulating the balance between proliferation and apoptosis. The PI3K/AKT/mTOR signaling pathway is also associated with angiogenesis and inflammation. In diabetic retinopathy, phosphorylation of PI3K induces phosphorylation of AKT and mTOR, leading to increased expression of VEGF and the secretion of tumor necrosis factor-alpha (TNF-α) and IL-1, which accelerates the progression of diabetic retinopathy [99]. Research has shown that the PI3K/AKT/mTOR signaling pathway also affects hormone levels. These insights underscore the potential of targeting the PI3K/AKT/mTOR signaling pathway to modulate the pathogenesis of adenomyosis, opening up novel avenues for both research and therapeutic intervention.

### 6.2. JAK/STAT

Continuous activation of the Janus kinase/Signal Transducer and Activator of Transcription (JAK/STAT) signaling pathway is a classical pathway that is abnormally overexpressed in many cancers, driving tumor cell survival, proliferation, invasion, and metastasis [100]. Under normal circumstances, the JAK/STAT pathway is largely inactive. However, adenomyosis disrupts this equilibrium, where an increase in IL-6 levels leads to the activation of the JAK2/STAT3 pathway, fostering EMT and enhancing cellular invasion capabilities [101,102]. Similarly, the suppression of miR-141-3p further stimulates the JAK2/STAT3 pathway [101], contributing to condition development. These insights underscore the potential of targeting the JAK2/STAT3 pathway—both its upstream and downstream components—as a strategy to slow adenomyosis progression.

### 6.3. HIPPO/YAP

**Figure 3:**
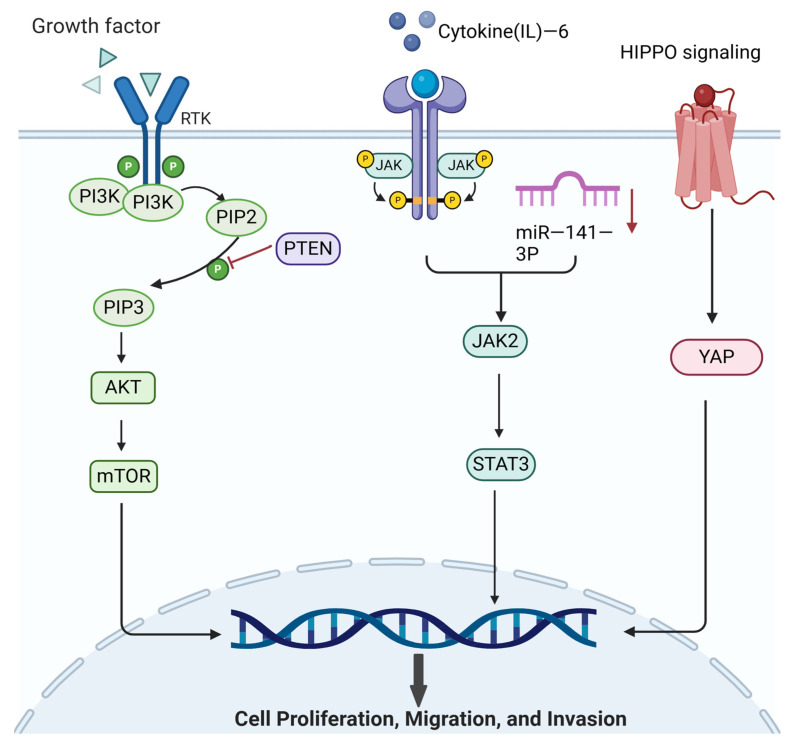
Migration and invasion signaling pathways. Created in BioRender. Hy, Z. (2024) https://BioRender.com/f11e869 (accessed on 31 October 2024). Legend: the red downward arrows indicate a decrease in the variable, while the red lines represent inhibitory effects.

Activation of the Hippo signaling pathway under physiological conditions can inhibit cell proliferation and migration and EMT and promote cell apoptosis. Yes-associated protein (YAP), a key downstream protein of the Hippo pathway, plays an important role in regulating cell proliferation and apoptosis and tissue metabolism [103]. YAP inhibitors have confirmed that inactivation of the Hippo signaling pathway in adenomyosis promotes proliferation, migration, and EMT occurrence [104]. By specifically targeting YAP1, researchers have observed improvements in progesterone resistance and an increase in PR expression, marking a significant advancement in adenomyosis treatment strategies [105]. Undoubtedly, the upstream and downstream molecules of the PI3K/AKT/mTOR, JAK/STAT, and Hippo signaling pathways are potential targets for adenomyosis treatment (Figure 3). However, experiments targeting these therapeutic strategies have focused on preclinical animal models; high-quality clinical trials are needed to support their clinical application.

## 7. Summary

**Figure 4:**
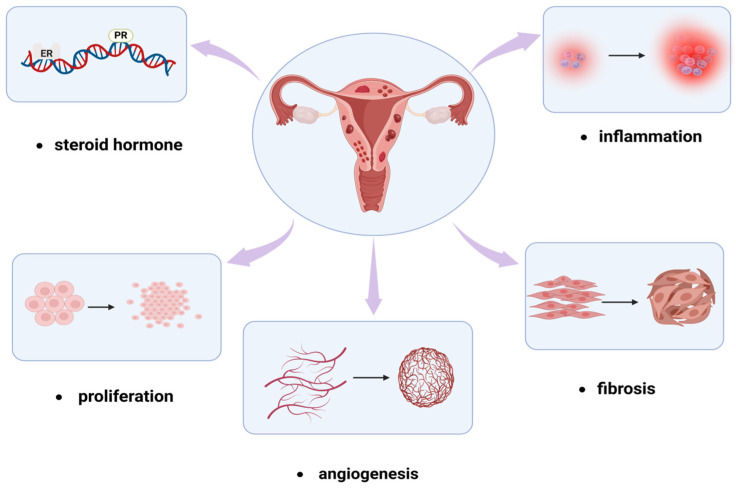
The pathophysiology of adenomyosis and representative pathways. Created in BioRender. Hy, Z. (2024) https://BioRender.com/m76z240 (accessed on 31 October 2024).

**Figure 5:**
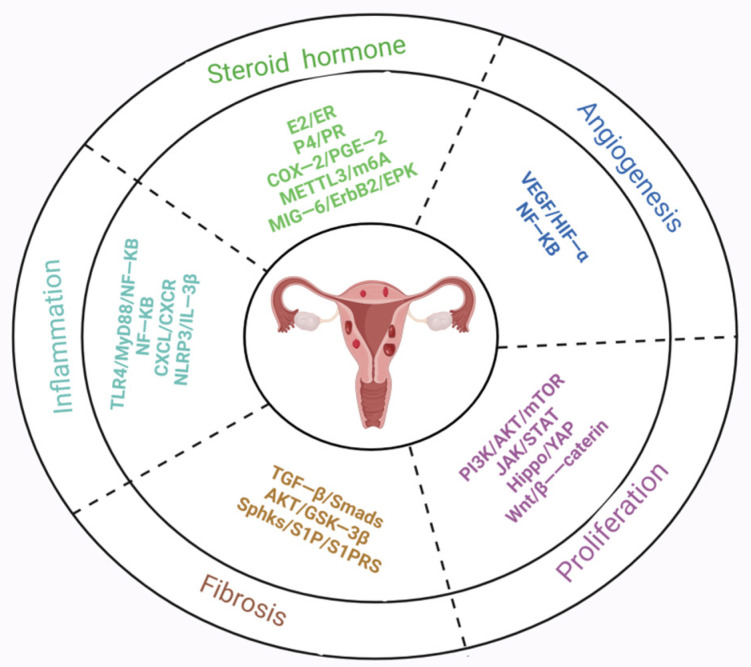
Cell signaling pathways participating in regulating pathological processes of adenomyosis. Created in BioRender. Hy, Z. (2024) https://BioRender.com/r27b722 (accessed on 31 October 2024).

The pathogenesis of adenomyosis is not yet fully understood, and its pathophysiological mechanisms are complex, making treatment a significant challenge. As abnormal signaling pathways play a crucial role in the pathogenesis of adenomyosis, targeting upstream and downstream effector molecules in these pathways emerges as a promising treatment strategy for the future. In conclusion, future research should focus on stable molecular signaling pathways and regulatory targets (Figure 4 and Figure 5), further exploring the complex interplay among signaling pathways that mediate the occurrence of adenomyosis lesions. Delving deeper into these interactions to uncover potential therapeutic targets can provide precise directions for the development of new drug treatments. However, it is essential to explore therapeutic strategies through multiple molecular pathways, as considering the role of a single signaling pathway in pathophysiology is limited and may adversely affect the overall function of the organism.

## 8. Conclusions

In conclusion, the transduction and regulation of signaling pathways is critical for the pathological development and targeted therapy of adenomyosis.

## Figures and Tables

**Table 1:** Cytokines changes in adenomyosis. (The arrows in the table indicate the changes in cytokine expression: upward arrows represent an increase in expression levels, while downward arrows represent a decrease in expression levels).

Type of Cytokines	Change	Pertinence	Type of Samples	Year
CD-68, IL-6, MCP-1	↑	endometrium receptivity	endometrium tissue	2016 [47]
IL-10	↓		endometrium tissue	2016 [47]
IL-10	↓	HOXA10	endometrium tissue	2018 [48]
IL23, IL31, IL25, IL33	↓	immunotolerant	serum	2019 [49]
TGF-β1	↑		uterine tissue	2019 [50]
IL10, IL22	↑	anti-inflammatory	uterine tissues	2021 [51]
IL6, IL1β, IFNα, TNF-α, IFNγ	↑	anti-inflammatory	uterine tissues	2021 [51]
IL-33	↓	HOXA10	uterine tissues	2022 [52]
IL-18			endometrium tissue	2022 [53]
IL-18BP	↓	invasion	endometrium	2022 [53]
CD163, IL10, TNF-α	↑	EMT	uterine tissues	2023 [54]
CCL26, CCL3, IL-1β	↑	EMT	endometrium tissue	2023 [55]
MCP-1, CXCR1, IL-1β	↑	pro-inflammatory	endometrium tissue	2023 [56]
IL-6, IFN-α	↑	pro-inflammatory	endometrium tissue	2024 [57]

## References

[1] Benagiano G., Lippi D., Habiba M., Oral E. (2022). A History of Adenomyosis. Endometriosis and Adenomyosis: Global Perspectives Across the Lifespan.

[2] Bird C.C., McElin T.W., Manalo-Estrella P. (1972). The elusive adenomyosis of the uterus–revisited. Am. J. Obstet. Gynecol..

[3] Chu L.H., Liao C.C., Liew P.L., Chen C.W., Su P.H., Wen K.C., Lai H.C., Huang R.L., Chen L.Y. (2022). Epigenomic Analysis Reveals the KCNK9 Potassium Channel as a Potential Therapeutic Target for Adenomyosis. Int. J. Mol. Sci..

[4] Moawad G., Fruscalzo A., Youssef Y., Kheil M., Tawil T., Nehme J., Pirtea P., Guani B., Afaneh H., Ayoubi J.M. (2023). Adenomyosis: An Updated Review on Diagnosis and Classification. J. Clin. Med..

[5] Harmsen M.J., Trommelen L.M., de Leeuw R.A., Tellum T., Juffermans LJ M., Griffioen A.W., Thomassin-Naggara I., Van den Bosch T., Huirne J.A.F. (2023). Uterine junctional zone and adenomyosis: Comparison of MRI, transvaginal ultrasound and histology. Ultrasound Obstet. Gynecol..

[6] Stratopoulou C.A., Donnez J., Dolmans M.M. (2021). Conservative Management of Uterine Adenomyosis: Medical vs. Surgical Approach. J. Clin. Med..

[7] Guo S.W. (2022). Cracking the enigma of adenomyosis: An update on its pathogenesis and pathophysiology. Reproduction.

[8] Inoue S., Hirota Y., Ueno T., Fukui Y., Yoshida E., Hayashi T., Kojima S., Takeyama R., Hashimoto T., Kiyono T. (2019). Uterine adenomyosis is an oligoclonal disorder associated with KRAS mutations. Nat. Commun..

[9] Boizet-Bonhoure B., Déjardin S., Girard M., Durix Q., Poulat F., Philibert P. (2024). Adenomyotic Lesions Are Induced in the Mouse Uterus after Exposure to NSAID and EE2 Mixtures at Environmental Doses. Int. J. Mol. Sci..

[10] Kok V.C., Tsai H.J., Su C.F., Lee C.K. (2015). The Risks for Ovarian, Endometrial, Breast, Colorectal, and Other Cancers in Women With Newly Diagnosed Endometriosis or Adenomyosis: A Population-Based Study. Int. J. Gynecol. Cancer.

[11] Vannuccini S., Clemenza S., Rossi M., Petraglia F. (2021). Hormonal treatments for endometriosis: The endocrine background. Rev. Endocr. Metab. Disord..

[12] Sztachelska M., Ponikwicka-Tyszko D., Martínez-Rodrigo L., Bernaczyk P., Palak E., Półchłopek W., Bielawski T., Wołczyński S. (2022). Functional Implications of Estrogen and Progesterone Receptors Expression in Adenomyosis, Potential Targets for Endocrinological Therapy. J. Clin. Med..

[13] Cozzolino M., Alsbjerg B., Pellicer A., Garcia-Velasco J.A., Humaidan P. (2024). The adenomyosis/endometriosis IVF patient—Call for clinical focus. Reprod. Biomed. Online.

[14] Yoo J.Y., Ku B.J., Kim T.H., Il Ahn J., Ahn J.Y., Yang W.S., Lim J.M., Taketo M.M., Shin J.H., Jeong J.W. (2020). β-catenin activates TGF-β-induced epithelial-mesenchymal transition in adenomyosis. Exp. Mol. Med..

[15] Zhang J., Shi L., Duan J., Li M., Li C. (2024). Proteomic detection of COX-2 pathway-related factors in patients with adenomyosis. PeerJ.

[16] Li C., Chen R., Jiang C., Chen L., Cheng Z. (2019). Correlation of LOX-5 and COX-2 expression with inflammatory pathology and clinical features of adenomyosis. Mol. Med. Rep..

[17] Jin Z., Wu X., Liu H., Xu C. (2020). Celecoxib, a selective COX-2 inhibitor, markedly reduced the severity of tamoxifen-induced adenomyosis in a murine model. Exp. Ther. Med..

[18] Jiang C., Gong W., Chen R., Ke H., Qu X., Yang W., Cheng Z. (2018). RhoA/ROCK/ARHGAP26 signaling in the eutopic and ectopic endometrium is involved in clinical characteristics of adenomyosis. J. Int. Med. Res..

[19] Sun F.Q., Duan H., Wang S., Li J.J. (2015). 17β-Estradiol Induces Overproliferation in Adenomyotic Human Uterine Smooth Muscle Cells of the Junctional Zone Through Hyperactivation of the Estrogen Receptor-Enhanced RhoA/ROCK Signaling Pathway. Reprod. Sci..

[20] Huang Z.X., Mao X.M., Wu R.F., Huang S.M., Ding X.Y., Chen Q.H., Chen Q.X. (2020). RhoA/ROCK pathway mediates the effect of oestrogen on regulating epithelial-mesenchymal transition and proliferation in endometriosis. J. Cell Mol. Med..

[21] Bulun S.E., Yildiz S., Adli M., Chakravarti D., Parker J.B., Milad M., Yang L., Chaudhari A., Tsai S., Wei J.J. (2023). Endometriosis and adenomyosis: Shared pathophysiology. Fertil. Steril..

[22] Kim T.H., Yoo J.-Y., Kim H.I., Gilbert J., Ku B.J., Li J., Mills G.B., Broaddus R.R., Lydon J.P., Lim J.M. (2014). Mig-6 Suppresses Endometrial Cancer Associated with Pten Deficiency and ERK Activation. Cancer Res..

[23] Yoo J.-Y., Kim T.H., Shin J.-H., Marquardt R.M., Müller U., Fazleabas A.T., Young S.L., Lessey B.A., Yoon H.-G., Jeong J.-W. (2022). Loss of MIG-6 results in endometrial progesterone resistance via ERBB2. Nat. Commun..

[24] Porru M., Pompili L., Caruso C., Biroccio A., Leonetti C. (2018). Targeting KRAS in metastatic colorectal cancer: Current strategies and emerging opportunities. J. Exp. Clin. Cancer Res..

[25] McCormick F. (2015). KRAS as a Therapeutic Target. Clin. Cancer Res..

[26] Chao A., Wu R.C., Lin C.Y., Lee L.Y., Tsai C.L., Lee Y.S., Wang C.J. (2023). Targeted next-generation sequencing for the detection of cancer-associated somatic mutations in adenomyosis. J. Obstet. Gynaecol..

[27] Wilson M.R., Reske J.J., Holladay J., Wilber G.E., Rhodes M., Koeman J., Adams M., Johnson B., Su R.-W., Joshi N.R. (2019). ARID1A and PI3-kinase pathway mutations in the endometrium drive epithelial transdifferentiation and collective invasion. Nat. Commun..

[28] Huang E., Chen L. (2023). RNA N(6)-methyladenosine modification in female reproductive biology and pathophysiology. Cell Commun. Signal..

[29] Zhai J., Li S., Sen S., Opoku-Anane J., Du Y., Chen Z.J., Giudice L.C. (2020). m(6)A RNA Methylation Regulators Contribute to Eutopic Endometrium and Myometrium Dysfunction in Adenomyosis. Front. Genet..

[30] Wan S., Sun Y., Zong J., Meng W., Yan J., Chen K., Wang S., Guo D., Xiao Z., Zhou Q. (2023). METTL3-dependent m(6)A methylation facilitates uterine receptivity and female fertility via balancing estrogen and progesterone signaling. Cell Death Dis..

[31] Zheng Z.H., Zhang G.L., Jiang R.F., Hong Y.Q., Zhang Q.Y., He J.P., Liu X.R., Yang Z.S., Yang L., Jiang X. (2023). METTL3 is essential for normal progesterone signaling during embryo implantation via m(6)A-mediated translation control of progesterone receptor. Proc. Natl. Acad. Sci. USA.

[32] Liu J., Eckert M.A., Harada B.T., Liu S.M., Lu Z., Yu K., Tienda S.M., Chryplewicz A., Zhu A.C., Yang Y. (2018). m(6)A mRNA methylation regulates AKT activity to promote the proliferation and tumorigenicity of endometrial cancer. Nat. Cell Biol..

[33] Włodarczyk M., Ciebiera M., Nowicka G., Łoziński T., Ali M., Al-Hendy A. (2024). Epigallocatechin Gallate for the Treatment of Benign and Malignant Gynecological Diseases-Focus on Epigenetic Mechanisms. Nutrients.

[34] Chen Y., Zhu B., Zhang H., Ding D., Liu X., Guo S.W. (2014). Possible Loss of GABAergic Inhibition in Mice With Induced Adenomyosis and Treatment With Epigallocatechin-3-Gallate Attenuates the Loss With Improved Hyperalgesia. Reprod. Sci..

[35] Chen Y., Zhu B., Zhang H., Liu X., Guo S.W. (2013). Epigallocatechin-3-gallate reduces myometrial infiltration, uterine hyperactivity, and stress levels and alleviates generalized hyperalgesia in mice induced with adenomyosis. Reprod. Sci..

[36] Strieter R.M., Burdick M.D., Mestas J., Gomperts B., Keane M.P., Belperio J.A. (2006). Cancer CXC chemokine networks and tumour angiogenesis. Eur. J. Cancer.

[37] Zhai J., Li S., Sen S., Vallvé-Juanico J., Irwin J.C., Vo K.C., Wan J., Du Y., Chen Z.J., Giudice L.C. (2022). Transcriptomic analysis supports collective endometrial cell migration in the pathogenesis of adenomyosis. Reprod. Biomed. Online.

[38] Janssens R., Struyf S., Proost P. (2018). The unique structural and functional features of CXCL12. Cell. Mol. Immunol..

[39] Cambier S., Gouwy M., Proost P. (2023). The chemokines CXCL8 and CXCL12: Molecular and functional properties, role in disease and efforts towards pharmacological intervention. Cell. Mol. Immunol..

[40] Liu X.N., Cheng Z.P. (2023). Expression of high-mobility group box-1 in eutopic/ectopic endometrium and correlations with inflammation-related factors in adenomyosis. Gynecol. Endocrinol..

[41] Ulukus M., Ulukus E.C., Seval Y., Cinar O., Zheng W., Arici A. (2006). Expression of interleukin-8 receptors in patients with adenomyosis. Fertil. Steril..

[42] Li W., Shao C., Liang J. (2024). Effects of Shixiao Huoxue Decoction on pain, tumor necrosis factor-α, and interleukin-8 in patients with adenomyosis. Am. J. Transl. Res..

[43] Nishimoto-Kakiuchi A., Sato I., Nakano K., Ohmori H., Kayukawa Y., Tanimura H., Yamamoto S., Sakamoto Y., Nakamura G., Maeda A. (2023). A long-acting anti-IL-8 antibody improves inflammation and fibrosis in endometriosis. Sci. Transl. Med..

[44] Tian J., Kang N., Wang J., Sun H., Yan G., Huang C., Mei J. (2022). Transcriptome analysis of eutopic endometrium in adenomyosis after GnRH agonist treatment. Reprod. Biol. Endocrinol..

[45] Wu X., Qian L., Zhao H., Lei W., Liu Y., Xu X., Li J., Yang Z., Wang D., Zhang Y. (2023). CXCL12/CXCR4: An amazing challenge and opportunity in the fight against fibrosis. Ageing Res. Rev..

[46] Harmsen M.J., Wong CF C., Mijatovic V., Griffioen A.W., Groenman F., Hehenkamp W.J.K., Huirne J.A.F. (2019). Role of angiogenesis in adenomyosis-associated abnormal uterine bleeding and subfertility: A systematic review. Hum. Reprod. Update.

[47] Zhihong N., Yun F., Pinggui Z., Sulian Z., Zhang A. (2016). Cytokine Profiling in the Eutopic Endometrium of Adenomyosis During the Implantation Window After Ovarian Stimulation. Reprod. Sci..

[48] Wang J., Huang C., Jiang R., Du Y., Zhou J., Jiang Y., Yan Q., Xing J., Hou X., Zhou J. (2018). Decreased Endometrial IL-10 Impairs Endometrial Receptivity by Downregulating HOXA10 Expression in Women with Adenomyosis. Biomed. Res. Int..

[49] Bourdon M., Santulli P., Chouzenoux S., Maignien C., Bailly K., Andrieu M., Millischer A.E., Doridot L., Marcellin L., Batteux F. (2019). The Disease Phenotype of Adenomyosis-Affected Women Correlates With Specific Serum Cytokine Profiles. Reprod. Sci..

[50] Cheong M.L., Lai T.H., Wu W.B. (2019). Connective tissue growth factor mediates transforming growth factor β-induced collagen expression in human endometrial stromal cells. PLoS ONE.

[51] Bourdon M., Santulli P., Jeljeli M., Vannuccini S., Marcellin L., Doridot L., Petraglia F., Batteux F., Chapron C. (2021). Immunological changes associated with adenomyosis: A systematic review. Hum. Reprod. Update.

[52] He B., Teng X.M., Hao F., Zhao M., Chen Z.Q., Li K.M., Yan Q. (2022). Decreased intracellular IL-33 impairs endometrial receptivity in women with adenomyosis. Front. Endocrinol..

[53] Chen L.H., Chan S.H., Li C.J., Wu H.M., Huang H.Y. (2022). Altered Expression of Interleukin-18 System mRNA at the Level of Endometrial Myometrial Interface in Women with Adenomyosis. Curr. Issues Mol. Biol..

[54] Hu Y., Yuan M., Cheng L., Wang G. (2023). Extracellular vesicles contribute to EMT in adenomyosis by inducing macrophage polarization^†^. Biol. Reprod..

[55] Ikebuchi A., Osaki M., Wada I., Nagata H., Nagira K., Azuma Y., Okada F., Harada T., Taniguchi F. (2023). Increased chemokine ligand 26 expression and its involvement in epithelial-mesenchymal transition in the endometrium with adenomyosis. J. Gynecol. Obstet. Hum. Reprod..

[56] Maclean A., Barzilova V., Patel S., Bates F., Hapangama D.K. (2023). Characterising the immune cell phenotype of ectopic adenomyosis lesions compared with eutopic endometrium: A systematic review. J. Reprod. Immunol..

[57] Wang K., Wen Y., Fu X., Wei S., Liu S., Chen M. (2024). mtDNA regulates cGAS-STING signaling pathway in adenomyosis. Free Radic. Biol. Med..

[58] Guo J., Chen L., Luo N., Li C., Chen R., Qu X., Liu M., Kang L., Cheng Z. (2016). LPS/TLR4-mediated stromal cells acquire an invasive phenotype and are implicated in the pathogenesis of adenomyosis. Sci. Rep..

[59] Mao C., Liu X., Guo S.W. (2023). Reduced endometrial expression of histone deacetylase 3 in women with adenomyosis who complained of heavy menstrual bleeding. Reprod. Biomed. Online.

[60] Wang Y., Jiao B., Hu Z., Wang Y. (2024). Critical Role of histone deacetylase 3 in the regulation of kidney inflammation and fibrosis. Kidney Int..

[61] Zhang L., Cao W. (2022). Histone deacetylase 3 (HDAC3) as an important epigenetic regulator of kidney diseases. J. Mol. Med..

[62] Kim T.H., Yoo J.Y., Choi K.C., Shin J.H., Leach R.E., Fazleabas A.T., Young S.L., Lessey B.A., Yoon H.G., Jeong J.W. (2019). Loss of HDAC3 results in nonreceptive endometrium and female infertility. Sci. Transl. Med..

[63] Feng T., Wei S., Wang Y., Fu X., Shi L., Qu L., Fan X. (2017). Rhein ameliorates adenomyosis by inhibiting NF-κB and β-Catenin signaling pathway. Biomed. Pharmacother..

[64] Liu L., Luo N., Guo J., Xie Y., Chen L., Cheng Z. (2018). Berberine inhibits growth and inflammatory invasive phenotypes of ectopic stromal cells: Imply the possible treatment of adenomyosis. J. Pharmacol. Sci..

[65] Park H., Kim S.H., Cho Y.M., Ihm H.J., Oh Y.S., Hong S.H., Chae H.D., Kim C.H., Kang B.M. (2016). Increased expression of nuclear factor kappa-B p65 subunit in adenomyosis. Obstet. Gynecol. Sci..

[66] Guan X., Liu D., Zhou H., Dai C., Wang T., Fang Y., Jia Y., Li K. (2022). Melatonin improves pregnancy outcomes in adenomyosis mice by restoring endometrial receptivity via NF-κB/apoptosis signaling. Ann. Transl. Med..

[67] Yao J., Sterling K., Wang Z., Zhang Y., Song W. (2024). The role of inflammasomes in human diseases and their potential as therapeutic targets. Signal Transduct. Target. Ther..

[68] Liu H., Zhao Y., Yang Y., Huang W., Chao L. (2022). GRIM19 downregulation-induced pyroptosis of macrophages through NLRP3 pathway in adenomyosis. Reprod. Biomed. Online.

[69] Dong W., Peng Q., Liu Z., Xie Z., Guo X., Li Y., Chen C. (2023). Estrogen plays an important role by influencing the NLRP3 inflammasome. Biomed. Pharmacother..

[70] Murakami M., Osuka S., Muraoka A., Hayashi S., Bayasula Kasahara Y., Sonehara R., Hariyama Y., Shinjo K., Tanaka H., Miyake N. (2022). Effectiveness of NLRP3 Inhibitor as a Non-Hormonal Treatment for ovarian endometriosis. Reprod. Biol. Endocrinol..

[71] Liu Y., Jiang Z., Zhang L., Tian W., Lin A., Li M. (2024). Blockage of the NLRP3 inflammasome by MCC950 inhibits migration and invasion in adenomyosis. Reprod. Biomed. Online.

[72] Niu W., Zhang Y., Liu H., Liang N., Xu L., Li Y., Yao W., Shi W., Liu Z. (2023). Single-Cell Profiling Uncovers the Roles of Endometrial Fibrosis and Microenvironmental Changes in Adenomyosis. J. Inflamm. Res..

[73] Liu X., Shen M., Qi Q., Zhang H., Guo S.W. (2016). Corroborating evidence for platelet-induced epithelial-mesenchymal transition and fibroblast-to-myofibroblast transdifferentiation in the development of adenomyosis. Hum. Reprod..

[74] Peng D., Fu M., Wang M., Wei Y., Wei X. (2022). Targeting TGF-β signal transduction for fibrosis and cancer therapy. Mol. Cancer.

[75] Song Y., Wei J., Li R., Fu R., Han P., Wang H., Zhang G., Li S., Chen S., Liu Z. (2023). Tyrosine kinase receptor B attenuates liver fibrosis by inhibiting TGF-β/SMAD signaling. Hepatology.

[76] Jacobo A., Borges R.F., de Souza CA B., Genro V.K., Cunha-Filho J.S. (2024). Transforming growth factor beta-1 (TGF-β1) expression in patients with adenomyosis. Rev. Bras. Ginecol. Obstet..

[77] Huang Y., Zheng W., Mu L., Ren Y., Chen X., Liu F. (2011). Expression of tyrosine kinase receptor B in eutopic endometrium of women with adenomyosis. Arch. Gynecol. Obstet..

[78] Zheng H., Yang Z., Xin Z., Yang Y., Yu Y., Cui J., Liu H., Chen F. (2020). Glycogen synthase kinase-3β: A promising candidate in the fight against fibrosis. Theranostics.

[79] Zhang K., Zhou Z., Wang C., Yu M., Zhang Y., Shi Y., Wang X., Liu Y., Xu L., Shi W. (2022). Mechanism Study of Cinnamomi Ramulus and Paris polyphylla Sm. Drug Pair in the Treatment of Adenomyosis by Network Pharmacology and Experimental Validation. Evid. Based Complement. Altern. Med..

[80] Bernacchioni C., Ciarmela P., Vannuzzi V., Greco S., Vannuccini S., Malentacchi F., Pellegrino P., Capezzuoli T., Sorbi F., Cencetti F. (2021). Sphingosine 1-phosphate signaling in uterine fibroids: Implication in activin A pro-fibrotic effect. Fertil. Steril..

[81] Di Paolo A., Vignini A., Alia S., Membrino V., Delli Carpini G., Giannella L., Ciavattini A. (2022). Pathogenic Role of the Sphingosine 1-Phosphate (S1P) Pathway in Common Gynecologic Disorders (GDs): A Possible Novel Therapeutic Target. Int. J. Mol. Sci..

[82] Bernacchioni C., Capezzuoli T., Vannuzzi V., Malentacchi F., Castiglione F., Cencetti F., Ceccaroni M., Donati C., Bruni P., Petraglia F. (2021). Sphingosine 1-phosphate receptors are dysregulated in endometriosis: Possible implication in transforming growth factor β–induced fibrosis. Fertil. Steril..

[83] Ader I., Malavaud B., Cuvillier O. (2009). When the Sphingosine Kinase 1/Sphingosine 1-Phosphate Pathway Meets Hypoxia Signaling: New Targets for Cancer Therapy. Cancer Res..

[84] Bernacchioni C., Rossi M., Vannuzzi V., Prisinzano M., Seidita I., Raeispour M., Muccilli A., Castiglione F., Bruni P., Petraglia F. (2023). Sphingosine-1-phosphate receptor 3 is a non-hormonal target to counteract endometriosis-associated fibrosis. Fertil. Steril..

[85] Vannuzzi V., Bernacchioni C., Muccilli A., Castiglione F., Nozzoli F., Vannuccini S., Capezzuoli T., Ceccaroni M., Bruni P., Donati C. (2022). Sphingosine 1-phosphate pathway is dysregulated in adenomyosis. Reprod. BioMed. Online.

[86] Lai T.H., Wu P.H., Wu W.B. (2016). Involvement of NADPH oxidase and NF-κB activation in CXCL1 induction by vascular endothelial growth factor in human endometrial epithelial cells of patients with adenomyosis. J. Reprod. Immunol..

[87] Huang T.S., Chen Y.J., Chou T.Y., Chen C.Y., Li H.Y., Huang B.S., Tsai H.W., Lan H.Y., Chang C.H., Twu N.F. (2014). Oestrogen-induced angiogenesis promotes adenomyosis by activating the Slug-VEGF axis in endometrial epithelial cells. J. Cell Mol. Med..

[88] Liang S., Shi L.Y., Duan J.Y., Liu H.H., Wang T.T., Li C.Y. (2021). Celecoxib reduces inflammation and angiogenesis in mice with adenomyosis. Am. J. Transl. Res..

[89] Yalaza C., Canacankatan N., Gürses İ., Aytan H., Taşdelen B. (2020). Altered VEGF, Bcl-2 and IDH1 expression in patients with adenomyosis. Arch. Gynecol. Obstet..

[90] Middelkoop M.A., Don E.E., Hehenkamp WJ K., Polman N.J., Griffioen A.W., Huirne J.A.F. (2023). Angiogenesis in abnormal uterine bleeding: A narrative review. Hum. Reprod. Update.

[91] Valdés G., Erices R., Chacón C., Corthorn J. (2008). Angiogenic, hyperpermeability and vasodilator network in utero-placental units along pregnancy in the guinea-pig (*Cavia porcellus*). Reprod. Biol. Endocrinol..

[92] Wang H.S., Tsai C.L., Chang P.Y., Chao A., Wu R.C., Chen S.H., Wang C.J., Yen C.F., Lee Y.S., Wang T.H. (2018). Positive associations between upregulated levels of stress-induced phosphoprotein 1 and matrix metalloproteinase-9 in endometriosis/adenomyosis. PLoS ONE.

[93] Li Z., Wei J., Chen B., Wang Y., Yang S., Wu K., Meng X. (2023). The Role of MMP-9 and MMP-9 Inhibition in Different Types of Thyroid Carcinoma. Molecules.

[94] Lawrence M.S., Stojanov P., Mermel C.H., Robinson J.T., Garraway L.A., Golub T.R., Meyerson M., Gabriel S.B., Lander E.S., Getz G. (2014). Discovery and saturation analysis of cancer genes across 21 tumour types. Nature.

[95] Choi J., Jo M., Lee E., Hwang S., Choi D. (2017). Aberrant PTEN expression in response to progesterone reduces endometriotic stromal cell apoptosis. Reproduction.

[96] Xu X.Y., Zhang J., Qi Y.H., Kong M., Liu S.A., Hu J.J. (2018). Linc-ROR promotes endometrial cell proliferation by activating the PI3K-Akt pathway. Eur. Rev. Med. Pharmacol. Sci..

[97] Juárez-Barber E., Segura-Benítez M., Carbajo-García M.C., Bas-Rivas A., Faus A., Vidal C., Giles J., Labarta E., Pellicer A., Cervelló I. (2023). Extracellular vesicles secreted by adenomyosis endometrial organoids contain miRNAs involved in embryo implantation and pregnancy. Reprod. Biomed. Online.

[98] Choi J., Jo M., Lee E., Lee D.Y., Choi D. (2015). Dienogest enhances autophagy induction in endometriotic cells by impairing activation of AKT, ERK1/2, and mTOR. Fertil. Steril..

[99] Guan R., Kang Z., Li L., Yan X., Gao T. (2024). PIK3CA regulates development of diabetes retinopathy through the PI3K/Akt/mTOR pathway. PLoS ONE.

[100] Mengie Ayele T., Tilahun Muche Z., Behaile Teklemariam A., Bogale Kassie A., Chekol Abebe E. (2022). Role of JAK2/STAT3 Signaling Pathway in the Tumorigenesis, Chemotherapy Resistance, and Treatment of Solid Tumors: A Systemic Review. J. Inflamm. Res..

[101] Wang S., Duan H., Wang S., Guo Z., Lin Q. (2023). miR-141-3p Regulates the Proliferation and Apoptosis of Endometrial-Myometrial Interface Smooth Muscle Cells in Adenomyosis Via JAK2/STAT3 Pathway. Biochem. Genet..

[102] Jiang X., Chen X. (2023). Endometrial cell-derived exosomes facilitate the development of adenomyosis via the IL-6/JAK2/STAT3 pathway. Exp. Ther. Med..

[103] Marti P., Stein C., Blumer T., Abraham Y., Dill M.T., Pikiolek M., Orsini V., Jurisic G., Megel P., Makowska Z. (2015). YAP promotes proliferation, chemoresistance, and angiogenesis in human cholangiocarcinoma through TEAD transcription factors. Hepatology.

[104] Jin T., Li M., Li T., Yan S., Ran Q., Chen W. (2023). The Inactivation of Hippo Signaling Pathway Promotes the Development of Adenomyosis by Regulating EMT, Proliferation, and Apoptosis of Cells. Reprod. Sci..

[105] Lin S.C., Li W.N., Lin S.C., Hou H.T., Tsai Y.C., Lin T.C., Wu M.H., Tsai S.J. (2023). Targeting YAP1 ameliorates progesterone resistance in endometriosis. Hum. Reprod..

